# Validation of a novel model for the early detection of hepatocellular carcinoma

**DOI:** 10.1186/s12014-018-9222-0

**Published:** 2019-01-16

**Authors:** Philip M. Hemken, Lori J. Sokoll, Xiaoqing Yang, Jianliang Dai, Debra Elliott, Susan H. Gawel, Michael Lucht, Ziding Feng, Jorge A. Marrero, Sudhir Srivastava, Daniel W. Chan, Gerard J. Davis

**Affiliations:** 1 Diagnostics Discovery Research and Development, Abbott Diagnostics, Abbott Laboratories, 100 Abbott Park Road, D09GP, AP20/1, Abbott Park, IL 60064 USA; 2 Division of Clinical Chemistry, Department of Pathology, The Johns Hopkins University, Baltimore, MD USA; 3 Division of Quantitative Sciences, Department of Biostatistics, The University of Texas, MD Anderson Cancer Center, Houston, TX USA; 4 Division of Digestive and Liver Diseases, University of Texas Southwestern, 5959 Harry Hines Blvd POB I Suite 420B, Dallas, TX USA; 5 Cancer Biomarkers Research Group, Division of Cancer Prevention, National Cancer Institute, Bethesda, MD USA; 6 Present Address: Novartis Institutes for BioMedical Research, Inc., Cambridge, MA USA

**Keywords:** Biomarker, Early diagnosis, Liver, Cancer, Des-gamma carboxyprothrombin

## Abstract

**Background:**

The biomarkers alpha-fetoprotein (AFP) and protein induced by vitamin K absence/antagonist-II (PIVKA-II) may be useful for detecting early-stage hepatocellular carcinoma (HCC). We evaluated the performance of AFP and PIVKA-II levels, alone and in combination with clinical factors, for the early detection of HCC.

**Methods:**

In a case–control study, serum AFP and PIVKA-II were measured using the ARCHITECT immunoassay analyzer system in a cohort of 119 patients with HCC, 215 patients with non-malignant liver disease, and 34 healthy subjects. Five predictive models for detecting HCC were developed based on age, gender, AFP, and/or PIVKA-II levels; the best model was validated in an independent cohort of 416 patients with HCC and 412 control subjects with cirrhosis.

**Results:**

In both cohorts, AFP and PIVKA-II concentrations were higher in patients with HCC compared to healthy controls and patients with non-malignant liver disease. The model that combined AFP and PIVKA-II, age, and gender had the highest AUC of 0.95 (0.95, 95% CI 0.93–0.98), with a sensitivity of 93% and a specificity of 84% in the development cohort, and an AUC of 0.87 (95% CI 0.85–0.90), sensitivity of 74%, and specificity of 85% in the validation cohort. When limiting the validation cohort to only early-stage HCC, the AUC was 0.85 (95% CI 0.81–0.88), sensitivity was 70%, and specificity was 86%.

**Conclusions:**

Compared to each biomarker alone, the combination of AFP and PIVKA-II with age and gender improved the accuracy of detecting HCC and differentiating HCC from non-malignant liver disease.

**Electronic supplementary material:**

The online version of this article (10.1186/s12014-018-9222-0) contains supplementary material, which is available to authorized users.

## Background

Worldwide, liver cancer is the fifth most common cancer in men and ninth most common in women, and the majority of primary liver cancers are hepatocellular carcinoma (HCC) [1]. The incidence of HCC has tripled between 1975 and 2011 in the US, with nearly 40,000 new cases diagnosed in 2016, primarily due to a rise in hepatitis C virus (HCV)-induced cirrhosis [1, 2]. Liver cancer is also one of the most fatal cancers, with a 5-year survival rate of 17% in the US [2] and less than 20% globally [1]. The poor prognosis of HCC is in large part related to late-stage diagnosis, as symptoms do not appear until advanced stages when there are fewer effective treatment options.

The 5-year survival rate is approximately 3% in patients with metastatic HCC [3], compared to 31% in patients with localized disease [2]. Thus, a number of clinical practice guidelines [4–8] recommend screening of high-risk patients, such as those with cirrhosis, to detect early-stage tumors and initiate treatment to improve outcomes [9]. Surveillance primarily involves imaging, most commonly by ultrasound with or without alpha-fetoprotein (AFP) every 6 months, as recommended by the recent guidelines from the American Association for the Study of Liver Diseases (AASLD) [2, 4]. However, early diagnosis of HCC by ultrasound alone is complicated by underlying cirrhosis and may increase the potential harms of surveillance, with low sensitivity and a high false negative rate (60%) [10]. Further, ultrasound is operator-dependent and has relatively poor reproducibility.

Circulating biomarkers may provide additional diagnostic information to complement ultrasound findings and may be particularly helpful in detecting biochemical changes associated with malignancy in the liver prior to the formation of hepatic nodules [11]. AFP is a widely used, yet imperfect biomarker for detection of liver cancer [12]. AASLD guidelines suggest that adding AFP to ultrasound may improve detection of HCC in at-risk patients with cirrhosis [4].

The biomarkers protein induced by vitamin K absence/antagonist-II (PIVKA-II), also known as des-gamma carboxyprothrombin (DCP), and AFP-L3, a glycosylated form of AFP that is more specific to liver cancer, have been investigated as additional HCC biomarkers. Abnormal carboxylation of the anticoagulation factor prothrombin by vitamin K-dependent carboxylase occurs in malignant hepatocytes, leading to increased levels of circulating PIVKA-II in patients with HCC [11, 13]. Several studies have shown that PIVKA-II has a higher sensitivity and specificity than AFP for detecting HCC versus non-malignant liver diseases [14–16]. However, a large, multicenter National Cancer Institute (NCI) Early Detection Research Network (EDRN) study in 836 patients reported similar areas under the receiver operating characteristic curve (ROC AUC) of 0.83 (95% CI 0.80–0.85) for AFP and 0.81 (95% CI 0.78–0.84) for PIVKA-II for differentiating between HCC and cirrhosis [17]. The same study demonstrated that the combination of AFP and PIVKA-II increased the AUC, particularly for the detection of early-stage disease [17]. PIVKA-II has been used clinically as a biomarker for risk stratification of HCC, and is now included in biomarker panels for HCC surveillance in Japanese guidelines [6, 7].

In a preliminary study of AFP and novel biomarkers for HCC using AFP and PIVKA-II assays on the Abbott ARCHITECT *i*2000 system, we showed that PIVKA-II had the highest diagnostic accuracy for HCC [18]. In the current study, we further evaluated the performance of the ARCHITECT AFP and PIVKA-II assays, alone and in combination with clinical factors, for the detection of HCC in populations of patients with HCC in the US, including those with early-stage HCC and non-malignant liver disease. We further validated our findings in the NCI EDRN cohort [17].

## Methods

### Study design and serum samples

This was a retrospective case–control study measuring the biomarkers AFP and PIVKA-II in serum samples collected between 2003 and 2016 at the Johns Hopkins Medical Institutions (JHMI) in Baltimore, MD, from patients with HCC or chronic liver disease (cirrhosis and pre-cirrhotic stages) with viral or non-viral etiology, and healthy controls. Serum samples from patients with HCC were collected prior to treatment. The study was approved by the Johns Hopkins Medicine IRB. Additional serum samples obtained after consent from patients with liver cirrhosis at the University of Texas Southwestern Medical Center (UTSMC) in Dallas, TX, were analyzed at JHMI. For each serum sample, the following de-identified data was collected: age, gender, race/ethnicity, etiology of liver disease, and HCC stage based on the TNM staging system [19], if applicable. These samples were used to develop and train the HCC detection models (development cohort).

Additional samples from the NCI EDRN cohort (validation cohort) were used to validate the best model derived from the development cohort. Validation cohort samples were obtained from EDRN [17] through an agreement with NCI. The EDRN study included 836 subjects; of these, 828 were included in this analysis (416 HCC with cirrhosis and 412 controls with cirrhosis only) and the study was powered for detecting at least a 15% sensitivity difference for a new biomarker compared with the performance of AFP alone. For each serum sample, the following de-identified data was collected: age, gender, race/ethnicity, etiology of liver disease, and HCC stage based on the Barcelona Clinic Liver Cancer (BCLC) staging system [20]. A direct comparison of the cohorts based on the TNM and BCLC systems was not possible because BCLC includes clinical criteria other than size [21].

### Sample storage and assays

Serum samples were stored at approximately − 80 °C prior to analysis. US-approved AFP and ex-US‒approved PIVKA-II were measured using the ARCHITECT *i*2000 immunoassay analyzer (Abbott Laboratories, North Chicago, IL) per the manufacturer’s instructions [22, 23]. Each two-step sandwich immunoassay utilizes paramagnetic microparticles coated with either anti-AFP [24] or anti-PIVKA-II [25] antibodies and a chemiluminescent signal for the quantitative measurement of AFP or PIVKA-II in human serum and plasma. The performance characteristics for the ARCHITECT AFP and PIVKA-II assays are described in Table 1.

**Table 1:** ARCHITECT assay performance characteristics [22, 23]

Parameter	AFP assay	PIVKA-II assay
20-Day precision	Total within-laboratory %CV of ≤ 7.5%	Total within-laboratory %CV ≤ 8.6%
LOQ	2.0 ng/mL	5.06 mAU/mL
LoD	≤1.0 ng/mL	1.45 mAU/mL
Dilution Linearity	Within ± 1 ng/mL for samples < 10 ng/mL, ± 10 ng/mL for samples 10–2000 ng/mL	Within ± 10% for samples 20–30,000 mAU/mL
Range	2–2000 ng/mL	5.06–30,000 mAU/mL
Extended range with autodilution	1:10 autodilution to 20,000 ng/mL	1:10 autodilution to 300,000 mAU/mL
HAMA/RF and Interferences	Within ± 10% for HAMA/RF and potential interferents, no notable endogenous interferences observed	Within ± 10% for HAMA/RF and potential interferents, no notable endogenous interferences observed

*CV* coefficient of variation, *HAMA* human anti-mouse antibodies, *LoD* limit of detection, *LoQ* limit of quantitation, *RF* rheumatoid factor

### Statistical analysis

Biomarker concentrations were stratified by disease category and HCC stage. The probability of each biomarker to detect HCC was determined and Random Forest (RF) classification models were used to explore the best combination of biomarkers for the detection of HCC. RF uses a resampling method to create a large collection of de-correlated trees, and then averages them. With the RF method, the bias of the full model is equivalent to the bias of a single decision tree, but the variance is much lower due to the nature of averaging a large collection of trees [26].

All of the JHMI/UTSMC sample results, comprising the development cohort, were used to train the models. The response variable for the models was the binary HCC status (any stage HCC vs. non-HCC). Multiple RF models were developed by selecting different combinations of age, gender, and the two biomarkers as the classifiers. The best model was selected based on the combination of classifiers with the highest ROC AUC. The sensitivities (SEs) and specificities (SPs) were reported at cut-off points where the sum of the sensitivity (SE) and the specificity (SP) were maximized. The confidence intervals of AUCs and SEs/SPs were calculated based on the two-sided non-parametric method developed by Delong et al. [27].

The best, final model selected from the development cohort was assessed further. To evaluate the generalizability of the best model in a different population, an independent, blinded data set from the NCI EDRN study was used to validate model performance. The validation cohort serum samples had been previously run on a PIVKA-II sandwich immunoassay (Eisai Co, Tokyo, Japan) and an AFP immunoassay on a Wako automated system (Mountain View, CA) [17]. To address bias with the Wako and Abbott immunoassay platforms, both AFP and PIVKA-II values for the validation cohort were transformed to the ARCHITECT concentration scale as follows. One hundred EDRN matched samples (50 cases and 50 controls) were randomly selected and measured using the ARCHITECT system. The linear regression coefficients (intercept a and slope b) between the AFP and PIVKA-II values in natural log scale from the EDRN study [17] and the corresponding values measured with the ARCHITECT system were obtained for the 100 samples. The transformed values were determined by applying the regression coefficients to the AFP/PIVKA-II values in natural log scale of all validation cohort samples followed by exponential transformation. The transformed AFP/PIVKA-II values were then used for subsequent analyses.

All statistical analyses were performed using R 3.1.2 (The R Foundation for Statistical Computing).

## Results

### Patient demographics

The development cohort consisted of serum samples from 70 patients with stage 1 or 2 HCC, 49 patients with stage 3 or 4 HCC, and samples from 215 patients with non-malignant liver disease (40 of whom had cirrhosis) and 34 healthy subjects (Table 2). The mean age for patients in the HCC, non-malignant liver disease, and healthy control groups were 61.5, 49.5, and 58.9 years, respectively, with the majority of patients being Caucasian or African American.

**Table 2:** JHMI/UTSMC development cohort demographics (N = 368)

	HCC (n = 119)^a^			Non-malignant liver disease^a^ (n = 215)	Healthy controls (n = 34)
	Stage 1 (n = 30)	Stage 2 (n = 40)	Stage 3 and 4 (n = 49)		
Age (years); median (IQR)	61.5 (56.2–67.0)	61 (58.0–64.5)	60 (57.0–65.5)	54 (42.0–60.0)	60.5 (51.2–64.8)
Age range (years)	45–88	18–85	45–80	8–75	40–77
Sex (male:female) [%]	77:23	75:25	85:15	60:40	50:50
Ethnicity (%)					
Caucasian	53	55	52	47	68
African American	37	33	40	38	6
Hispanic/Latino	3	5	2	2	0
Asian	7	0	6	10	0
Native American/Pacific Islander	0	3	0	0	0
Other	0	0	0	1	0
Unknown	0	5	0	1	26
Etiology (%)					
Non-viral	27	28	29	–	–
HBV	7	0	6		
HCV	53	65	52		
HBV and HCV	0	5	4		
Unknown	13	2	8	–	–

^a^Chronic hepatitis (n = 102); fibrosis, pre-cirrhotic (n = 19); cirrhosis (n = 40); hepatitis with cirrhosis (n = 54)

Demographics of the validation cohort (n = 828) are shown in Table 3. The cohort included 416 patients with HCC, the majority of whom had BCLC stage A disease, and 412 subjects with cirrhosis. Patient age varied from 26 to 82 years, with a greater proportion of men in each group. The majority of patients were Caucasian or African American, and the majority had chronic hepatitis C (HCV) infection. The development and validation cohorts had similar demographics in terms of average age, ratio of men to women, and race/ethnicity distribution, and the majority of cases in both cohorts had a viral etiology.

**Table 3:** NCI EDRN validation cohort demographics (N = 828)

	BCLC (Cirrhosis with HCC; n = 416)					Controls (cirrhosis only) (n = 412)
	0 (n = 10)	A (n = 223)	B (n = 81)	C (n = 91)	D (n = 11)	
Age (years); mean (SD)	59.3 (9.1)	60.9 (10.4)	62.1 (9.5)	58.8 (9.7)	63.2 (9.3)	54.9 (8.7)
Age range (years)	45–77	37–86	36–82	26–80	49–75	25–82
Sex (male:female)	60:40	74:26	91:9	86:14	73:27	70:30
Ethnicity (%)						
Caucasian	40	54	68	77	73	79
African-American	50	11	2	9	9	4
Asian	0	23	24	9	9	7
American Indian or Alaska native	0	9	2	3	0	8
Unknown/refused	10	3	4	2	9	2
Etiology (%)						
Alcoholic	0	10.8	14.8	8.8	0	11.6
HBV	0	20.6	13.6	9. 9	0	5.6
HCV	80	50.7	54.3	50.6	63.6	58.2
Others	20	17.9	17.3	30.8	36.4	24.5

### Biomarker concentrations

**Fig. 1:**
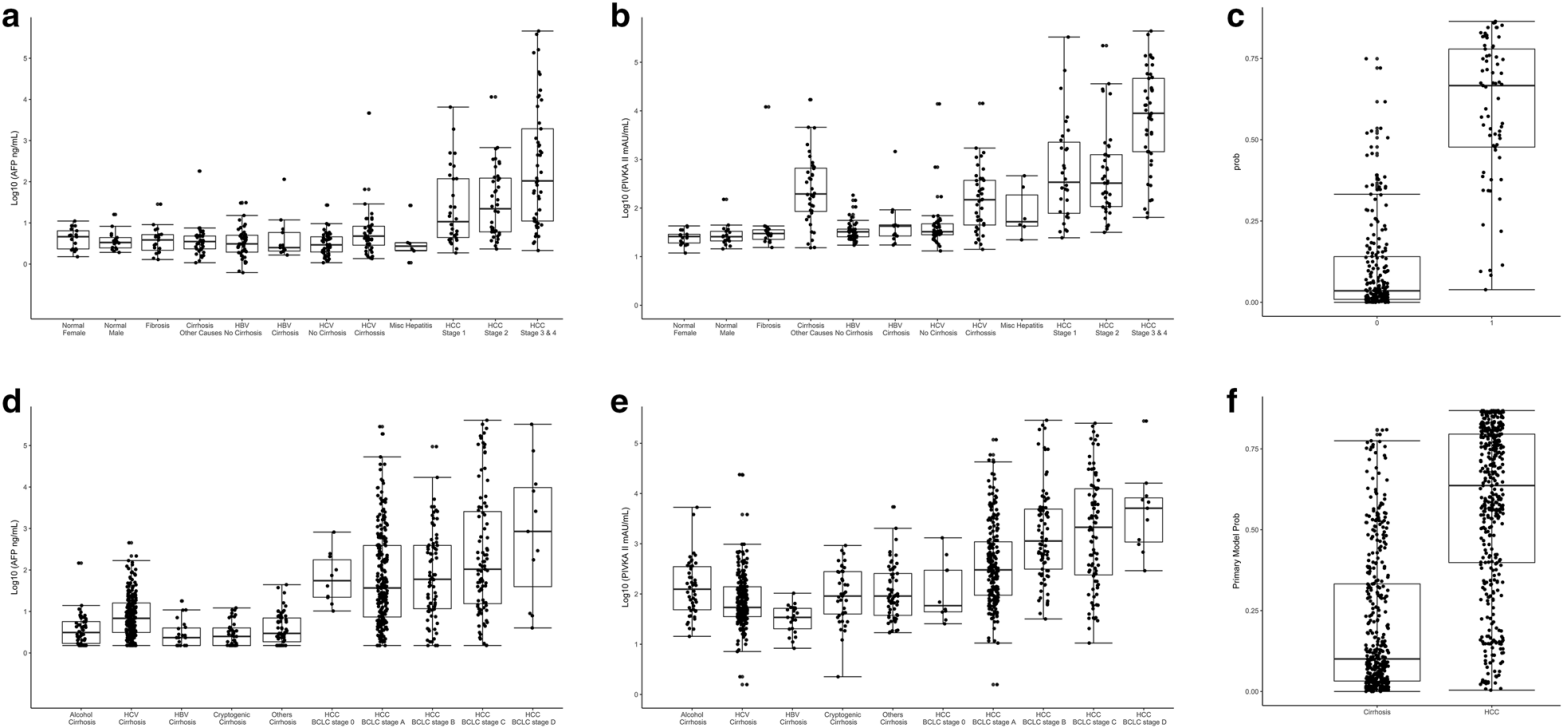
Concentrations of biomarkers for each study subject in the development cohort (JHMI/UTSMC) and validation cohort (NCI EDRN). **a** AFP concentration in the development cohort; **b** PIVKA-II concentration in the development cohort; **c** probability of HCC detection in the development cohort (no cancer = 0, cancer = 1); **d** AFP concentration in the validation cohort; (E) PIVKA-II concentration in the validation cohort; **f** probability of HCC detection in the validation cohort (cirrhosis vs. cancer)

In the development cohort, AFP and PIVKA-II concentrations were found to be higher in patients with HCC than in healthy controls (p < 0.0010) and patients with chronic liver disease (p < 0.0010), with levels increasing with HCC stage (Fig. 1a, b; Additional file 1: Table S1). AFP and PIVKA-II generally demonstrated higher levels in the HCC groups than in the non-HCC and control groups, and showed increasing levels with increasing stages of HCC, as shown in a probability plot (Fig. 1c). In the validation cohort, AFP and PIVKA-II levels yielded similar patterns as seen in the development cohort (Fig. 1d, e), and the probability of the biomarker associated with the presence and staging of HCC is shown in Fig. 1f.

The AFP and PIVKA-II concentrations in the validation cohort significantly correlated with the concentrations in the development cohort based on 50 control/50 HCC samples (AFP Spearman correlation coefficient ρ = 0.933, p < 0.0001; PIVKA-II Spearman correlation coefficient ρ = 0.826, p < 0.0001). However, Passing-Bablok regression and Deming regression showed systematic differences between two cohorts (data not shown). Therefore, a linear transformation method was employed to remove the systematic bias between the two cohorts.

### Model performance in the development cohort

**Fig. 2:**
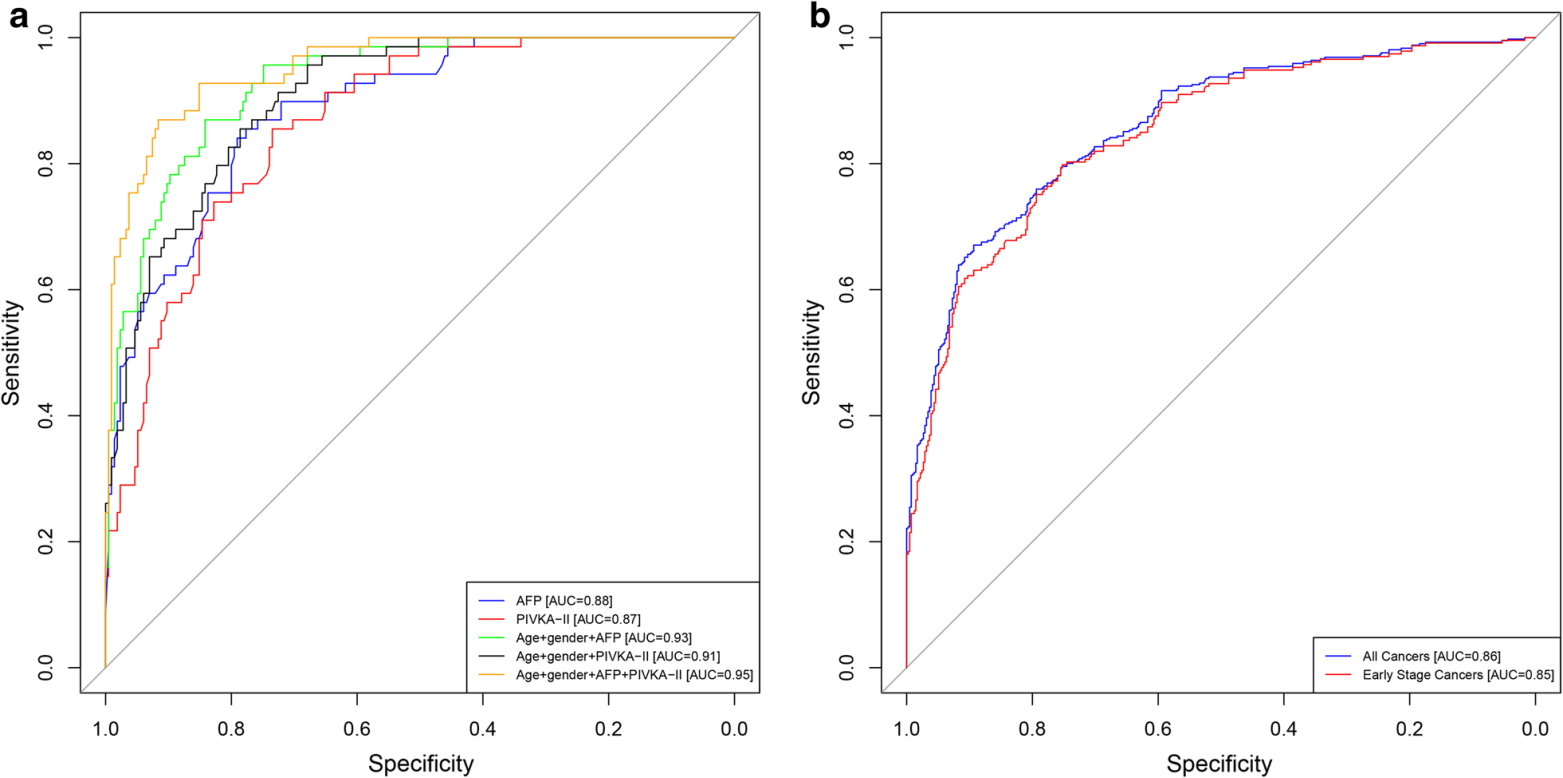
ROC analysis. **a** ROC for the development cohort (JHMI/UTSMC) for AFP (blue), PIVKA-II (red), age + gender + AFP (green), age + gender + PIVKA-II (black), age + gender + AFP + PIVKA-II (brown); **b** ROC for the validation cohort (EDRN) for age + gender + AFP + PIVKA-II for all cancers (blue) and for early-stage cancers (BCLC stage 0 and A; red)

Five models were developed based on the development cohort data using age, gender, AFP, and/or PIVKA-II, and the performance of these five models were compared to each other (described in Methods). The AUC for differentiating HCC from non-malignant liver disease was similar for AFP alone (Model 1: 0.88, 95% CI 0.84–0.93) and PIVKA-II alone (Model 2: 0.87, 95% CI 0.82–0.90) (Fig. 2a). The addition of age and gender to either AFP or PIVKA-II increased the AUCs (Model 3: 0.93, 95% CI 0.90–0.96 and Model 4: 0.91, 95% CI 0.87–0.94, respectively), but the increases were not statistically significant. The best model included a combination of both biomarkers, age, and gender, which increased the AUC to 0.95 (Model 5: 95% CI 0.93–0.98), with a sensitivity of 93% and a specificity of 84%. The increase was statistically significant compared to AFP or PIVKA-II alone and either biomarker combined with age and gender (p values between 0.0000 and 0.0042). The combination of either biomarker with age and gender increased sensitivity, with only a small decrease in specificity (Table 4). When specificity was held to 90%, sensitivity reached 84%.

**Table 4:** Diagnostic performance of biomarkers alone and in combination with clinical factors in the development cohort (JHMI/UTSMC) and in the model 5 in the validation cohort (NCI EDRN)

Model	Predictor variables		AUC	AUC 95% CI	SE	SP	SE (SP = 0.90)	SP (SE = 0.90)	SP (SE = 0.75)
Development cohort									
1	AFP		0.88	0.84–0.93	0.86	0.77	0.64	0.64	0.81
2	PIVKA-II		0.87	0.82–0.90	0.86	0.72	0.51	0.65	0.77
3	Age, gender, AFP		0.93	0.90–0.96	0.94	0.76	0.78	0.78	0.91
4	Age, gender, PIVKA-II		0.91	0.87–0.94	0.93	0.72	0.67	0.72	0.86
5	Age, gender, AFP, PIVKA-II		0.95	0.93–0.98	0.93	0.84	0.84	0.83	0.97
Validation cohort									
5^a^	Age, gender, AFP, PIVKA-II	All	0.87	0.85–0.90	0.74	0.85	0.67	0.54	0.84
		Viral etiology	0.86	0.83–0.89	0.79	0.80	0.62	0.61	0.82
		Non-viral etiology	0.87	0.83–0.91	0.75	0.91	0.75	0.58	0.91
5^b^	Age, gender, AFP, PIVKA-II	All	0.85	0.81–0.88	0.70	0.86	0.63	0.51	0.79
		Viral etiology	0.86	0.82–0.89	0.81	0.80	0.60	0.61	0.83
		Non-viral etiology	0.83	0.77–0.90	0.68	0.91	0.68	0.57	0.74

^a^All cancers

^b^Early-stage cancers (BCLC stage 0 and A)

### Model validation in the EDRN cohort

The best model from the development cohort (model 5) was evaluated using the validation cohort data as an independent assessment of clinical performance (Table 4). Model 5, which combines both biomarkers, age, and gender, had an estimated AUC of 0.87 (95% CI 0.85–0.90) in the validation cohort. Model 5 had an estimated sensitivity of 74% and a specificity of 85%; when specificity was held to 90%, sensitivity was estimated to be 67%. When limiting the validation cohort to only early-stage HCC (BCLC stage 0 and A), the estimated AUC was 0.85 (95% CI 0.81–0.88), sensitivity was 70%, and specificity was 86% for model 5. Model 5 was further assessed in the validation cohort stratified by non-viral and viral etiologies for all cancers and early-stage cancers. AUCs were comparable for viral and non-viral cancers, though the model had a slightly lower sensitivity and higher specificity for detecting all cancers and early-stage cancers with non-viral etiology compared to those with viral etiology.

## Discussion

We report here that the biomarkers AFP and PIVKA-II, when combined with age and gender, showed superior sensitivity and specificity for HCC detection compared to AFP and PIVKA-II alone or individually combined with age and gender. Further, analysis of the model in an independent validation cohort showed similar clinical performance, although with lower AUC, sensitivities, and specificities. This is important because the development cohort control group had a small number of patients with cirrhosis, while the validation cohort control group was comprised only of patients with cirrhosis. This study demonstrated the robustness of the HCC detection model with an external cohort dataset from a population of diverse composition.

Our findings are consistent with previous studies of the diagnostic accuracy of HCC biomarker panels in Asian and Western populations. In a prospective study of 734 high-risk Japanese patients with chronic hepatitis or liver cirrhosis, Ishii et al. [28] found that the combination of AFP and PIVKA-II had 65% sensitivity and 85% specificity for detecting early-stage HCC. A nested case–control study in China that included 45 patients with HCC and 138 matched controls found a similar increase in the diagnostic accuracy of the combination of AFP and PIVKA-II over either biomarker alone in patients with HCC [29]. In the US, the HALT-C trial reported that the sensitivity and specificity of PIVKA-II (74% and 46%) for the detection of early HCC were higher than those of AFP (61% and 81%), but the combination had a higher sensitivity than either biomarker alone (91%), with a 74% specificity [30]. The original EDRN case–control study of 419 US patients with HCC (208 early-stage) and 417 controls with cirrhosis found that AFP had a higher ROC AUC (0.80, 95% CI 0.77–0.84) than PIVKA-II (0.72, 95% CI 0.68–0.77), with the AUC of the combined biomarkers slightly higher than either biomarker alone (0.83, 95% CI 0.80–0.87) [17]. Our model 5 analyses in the validation cohort performed slightly better than the analyses in the original EDRN study (AUC = 0.87, 95% CI 0.85–0.90). No other biomarkers have shown better results.

One rationale for combining multiple biomarkers is that each may detect different aspects of early HCC tumor biology and provide additive information. One prospective study by Izuno et al. [31] reported that AFP was better able to detect small local tumors while PIVKA-II was more sensitive for detecting more diffuse tumors, with the combination of biomarkers having a higher diagnostic accuracy. Other studies have examined the addition of AFP-L3% as a third biomarker to improve accuracy; AFP-L3 is a glycosylated form of AFP that is specifically produced by HCC cells and has been shown to be better than AFP at differentiating between patients with HCC or cirrhosis [32]. In a study of 685 patients with HCC, 77% of patients had at least one elevated biomarker, the levels of AFP, PIVKA-II, and AFP-L3% correlated with the extent of disease as well as patient outcomes, and all three biomarkers decreased with treatment [33].

In this study, we found that PIVKA-II had lower specificity and similar sensitivity as AFP in the development cohort. Yu et al. also reported a consistently lower sensitivity of PIVKA-II compared with AFP, with similar specificities [29]; however, this is not seen consistently in the literature. Volk et al. reported that PIVKA-II is superior to either AFP or AFP-L3% at differentiating between HCC and cirrhosis (sensitivity 86%, specificity 93%), but that the AUC is lower for patients with high-risk HCC vs. low-risk HCC [34].

Differences between our findings and those of others may be related to the use of the RF model for statistical analysis, which does not specify a cut-off point, as well as differences in the patient population. When using AFP as a biomarker, a modified threshold that considers various factors, such as disease etiology/spectrum, underlying viral infection, age, and race/ethnicity for different populations may improve diagnostic accuracy [17, 35, 36]. For example, HCV is the causative agent largely responsible for the increase in incidence of HCC in the US; while HBV is the leading cause of HCC worldwide, particularly in Asia and Africa. Thus, taking viral infection into account when setting AFP biomarker thresholds may improve assessment of HCC risk in the US versus other countries.

A limitation of this study was that, given the retrospective nature of the analysis, the control groups in the development and validation cohorts were somewhat different in terms of composition and different staging systems were used, which limits assessment of specific confounders (Tables 2 and 3). A strength of this study was the demonstration of the robustness of the HCC model 5 with data from the external EDRN cohort. The large size of the validation cohort made it possible to examine the diagnostic performance of the model in subgroups of early-stage versus all cancers and viral versus non-viral etiology. The next step in the validation process is a phase 3 biomarker study using a prospective-specimen-collection, retrospective-blinded-evaluation (PRoBE) design [37], and such a study is currently underway.

## Conclusions

The use of a biomarker panel of AFP and PIVKA-II in combination with age and gender improved accuracy of detecting HCC and differentiating HCC from non-malignant liver disease in a US study population as compared to the individual biomarkers alone. Additional analyses are needed to assess the diagnostic accuracy of the AFP and PIVKA-II panel for early-stage vs later-stage HCC. Further validation in a phase 3 biomarker study is needed to support the use of multiple biomarker panels to aid in the early detection of HCC.

## Additional file

**Additional file 1: Table S1.** Median and Interquartile Range of AFP (ng/mL) and PIVKA-II (mAU/mL) Assay Results for Subjects in the Development (JHMI) and Validation (EDRN)* Cohorts, by Disease Category.

## Abbreviations

HCC: hepatocellular carcinoma
HCV: hepatitis C virus
HBV: hepatitis B virus
AASLD: American Association for the Study of Liver Diseases
AFP: alpha-fetoprotein
NCCN: National Comprehensive Cancer Network
NACB: National Academy of Clinical Biochemistry
PIVKA-II: protein induced by vitamin K absence/antagonist-II
DCP: des-gamma carboxyprothrombin
NCI: National Cancer Institute
EDRN: Early Detection Research Network
ROC: receiver operating curve
AUC: area under the curve
JHMI: Johns Hopkins Medical Institutions
UTSMC: University of Texas Southwestern Medical Center
BCLC: Barcelona Clinic Liver Cancer
RF: random forest
SE: sensitivity
SP: specificity
