# Supplementary material for: Validation of a novel model for the early detection of hepatocellular carcinoma
Source: Clin Proteomics. 2019 Jan 16;16:2. doi: 10.1186/s12014-018-9222-0 (PMC6334458; doi:10.1186/s12014-018-9222-0)
Supplement: Supplementary file 1 — Additional file 1: Table S1. Median and Interquartile Range of AFP (ng/mL) and PIVKA-II (mAU/mL) Assay Results for Subjects in the Development (JHMI) and Validation (EDRN)* Cohorts, by Disease Category. [file 12014_2018_9222_MOESM1_ESM.docx]

**Additional file 1: Table S1.** Median and Interquartile Range of AFP (ng/mL) and PIVKA-II (mAU/mL) Assay Results for Subjects in the Development (JHMI) and Validation (EDRN)* Cohorts, by Disease Category

| Cohort | Variable | Category | Median | Q1 | Q3 | IQR |
| --- | --- | --- | --- | --- | --- | --- |
| JHMI | AFP | Normal, Female | 4.62 | 2.33 | 6.43 | 4.1 |
| JHMI | AFP | Normal, Male | 3.35 | 2.47 | 4.4 | 1.93 |
| JHMI | AFP | Fibrosis | 3.86 | 2.18 | 5.2 | 3.03 |
| JHMI | AFP | Cirrhosis, Other Causes | 3.53 | 2.35 | 4.81 | 2.46 |
| JHMI | AFP | HBV, No Cirrhosis | 3.1 | 1.96 | 5.03 | 3.07 |
| JHMI | AFP | HBV, Cirrhosis | 2.52 | 2.07 | 5.85 | 3.78 |
| JHMI | AFP | HCV, No Cirrhosis | 2.93 | 1.97 | 4.6 | 2.63 |
| JHMI | AFP | HCV, Cirrhosis | 4.78 | 2.88 | 8.3 | 5.42 |
| JHMI | AFP | Misc. Hepatitis | 2.76 | 2.16 | 3.28 | 1.12 |
| JHMI | AFP | HCC, Stage 1 | 10.85 | 4.43 | 118.44 | 114.01 |
| JHMI | AFP | HCC, Stage 2 | 22.28 | 6.07 | 122.48 | 116.41 |
| JHMI | AFP | HCC, Stage 3 & 4 | 104.69 | 11.11 | 1932.02 | 1920.91 |
| JHMI | PIVKA-II | Normal, Female | 25.99 | 19.12 | 28.82 | 9.7 |
| JHMI | PIVKA-II | Normal, Male | 25.75 | 21.04 | 33.26 | 12.22 |
| JHMI | PIVKA-II | Fibrosis | 29.87 | 22.79 | 35.72 | 12.94 |
| JHMI | PIVKA-II | Cirrhosis, Other Causes | 195.28 | 85.45 | 661.57 | 576.12 |
| JHMI | PIVKA-II | HBV, No Cirrhosis | 32.46 | 25.63 | 36.82 | 11.19 |
| JHMI | PIVKA-II | HBV, Cirrhosis | 42.51 | 26.64 | 45.06 | 18.42 |
| JHMI | PIVKA-II | HCV, No Cirrhosis | 32.57 | 28.26 | 47.42 | 19.16 |
| JHMI | PIVKA-II | HCV, Cirrhosis | 148.53 | 44.8 | 372.02 | 327.22 |
| JHMI | PIVKA-II | Misc. Hepatitis | 52.78 | 42.56 | 218.28 | 175.72 |
| JHMI | PIVKA-II | HCC, Stage 1 | 344.15 | 77.98 | 2294.07 | 2216.09 |
| JHMI | PIVKA-II | HCC, Stage 2 | 325.45 | 107.51 | 1255.12 | 1147.62 |
| JHMI | PIVKA-II | HCC, Stage 3 & 4 | 8897.21 | 1444.68 | 46547.24 | 45102.56 |
| EDRN | AFP | Alcohol, Cirrhosis | 3.12 | 1.7 | 5.73 | 4.03 |
| EDRN | AFP | HCV, Cirrhosis | 6.83 | 3.12 | 16.06 | 12.95 |
| EDRN | AFP | HBV, Cirrhosis | 2.34 | 1.51 | 4.01 | 2.5 |
| EDRN | AFP | Cryptogenic, Cirrhosis | 2.5 | 1.51 | 4.01 | 2.5 |
| EDRN | AFP | Others, Cirrhosis | 2.96 | 1.86 | 6.97 | 5.11 |
| EDRN | AFP | HCC, BCLC stage 0 | 57.54 | 22.04 | 183.94 | 161.91 |
| EDRN | AFP | HCC, BCLC stage A | 36.86 | 7.38 | 390.3 | 382.93 |
| EDRN | AFP | HCC, BCLC stage B | 59.63 | 11.68 | 391.7 | 380.02 |
| EDRN | AFP | HCC, BCLC stage C | 104.29 | 15.45 | 2568.16 | 2552.7 |
| EDRN | AFP | HCC, BCLC stage D | 849 | 92.42 | 9896.2 | 9803.78 |
| EDRN | PIVKA.II | Alcohol, Cirrhosis | 124.94 | 48.55 | 349.99 | 301.44 |
| EDRN | PIVKA.II | HCV, Cirrhosis | 54.1 | 35.44 | 139.07 | 103.63 |
| EDRN | PIVKA.II | HBV, Cirrhosis | 34.26 | 20.29 | 51.84 | 31.56 |
| EDRN | PIVKA.II | Cryptogenic, Cirrhosis | 90.97 | 39.91 | 278.53 | 238.62 |
| EDRN | PIVKA.II | Others, Cirrhosis | 90.97 | 37.57 | 255.7 | 218.13 |
| EDRN | PIVKA.II | HCC, BCLC stage 0 | 59.36 | 44.2 | 297.23 | 253.03 |
| EDRN | PIVKA.II | HCC, BCLC stage A | 301.85 | 94.75 | 1094.15 | 999.4 |
| EDRN | PIVKA.II | HCC, BCLC stage B | 1134.16 | 312.71 | 4895.38 | 4582.67 |
| EDRN | PIVKA.II | HCC, BCLC stage C | 2127.68 | 239.87 | 12430.92 | 12191.05 |
| EDRN | PIVKA.II | HCC, BCLC stage D | 5096.68 | 1087.97 | 8219.62 | 7131.65 |

*Transformed data.
